# Sewage effluent from an Indian hospital harbors novel carbapenemases and integron-borne antibiotic resistance genes

**DOI:** 10.1186/s40168-019-0710-x

**Published:** 2019-06-27

**Authors:** Nachiket P. Marathe, Fanny Berglund, Mohammad Razavi, Chandan Pal, Johannes Dröge, Sharvari Samant, Erik Kristiansson, D. G. Joakim Larsson

**Affiliations:** 1 Centre for Antibiotic Resistance Research (CARe), University of Gothenburg, Gothenburg, Sweden; 2 Department of Infectious Diseases, Institute of Biomedicine, Sahlgrenska Academy, University of Gothenburg, Gothenburg, Sweden; 3 Institute of Marine Research (IMR), Bergen, Norway; 4 Department of Mathematical Sciences, Chalmers University of Technology and University of Gothenburg, Gothenburg, Sweden; 5 Plant Health and Environment Laboratory (PHEL), Ministry for Primary Industries (MPI), Auckland, New Zealand; 6 Mahatma Gandhi Mission medical college, Navi Mumbai, Maharashtra India

**Keywords:** Antibiotic resistance, Hospital effluent, Carbapenemase, Pathogens, Hidden Markov model, India

## Abstract

**Background:**

Hospital wastewaters contain fecal material from a large number of individuals, of which many are undergoing antibiotic therapy. It is, thus, plausible that hospital wastewaters could provide opportunities to find novel carbapenemases and other resistance genes not yet described in clinical strains. Our aim was therefore to investigate the microbiota and antibiotic resistome of hospital effluent collected from the city of Mumbai, India, with a special focus on identifying novel carbapenemases.

**Results:**

Shotgun metagenomics revealed a total of 112 different mobile antibiotic resistance gene types, conferring resistance against almost all classes of antibiotics. Beta-lactamase genes, including encoding clinically important carbapenemases, such as NDM, VIM, IMP, KPC, and OXA-48, were abundant. NDM (0.9% relative abundance to 16S rRNA genes) was the most common carbapenemase gene, followed by OXA-58 (0.84% relative abundance to 16S rRNA genes). Among the investigated mobile genetic elements, class 1 integrons (11% relative abundance to 16S rRNA genes) were the most abundant. The genus *Acinetobacter* accounted for as many as 30% of the total 16S rRNA reads, with *A. baumannii* accounting for an estimated 2.5%. High throughput sequencing of amplified integron gene cassettes identified a novel functional variant of an IMP-type (proposed IMP-81) carbapenemase gene (eight aa substitutions) along with recently described novel resistance genes like *sul4* and *bla*_RSA1_. Using a computational hidden Markov model, we detected 27 unique metallo-beta-lactamase (MBL) genes in the shotgun data, of which nine were novel subclass B1 genes, one novel subclass B2, and 10 novel subclass B3 genes. Six of the seven novel MBL genes were functional when expressed in *Escherichia coli.*

**Conclusion:**

By exploring hospital wastewater from India, our understanding of the diversity of carbapenemases has been extended. The study also demonstrates that the microbiota of hospital wastewater can serve as a reservoir of novel resistance genes, including previously uncharacterized carbapenemases with the potential to spread further.

**Electronic supplementary material:**

The online version of this article (10.1186/s40168-019-0710-x) contains supplementary material, which is available to authorized users.

## Background

The rise in antibiotic resistance is a serious growing problem for human health [1]. Environmental and commensal microbiota serves as sources for antibiotic resistance genes (ARGs) that emerge over time in pathogens through horizontal gene transfer [2, 3]. Characterization of the environmental resistome, thus, would provide an understanding of novel resistance factors that might be encountered in clinics in the future. This in turn would help to better understand the development of resistance in pathogens and to prepare surveillance and control measures to reduce their dissemination.

Recently, hidden Markov model (HMM)-based methods have been developed for detecting ARGs from shotgun sequence data [4–7]. We have successfully applied HMM to identify novel quinolone resistance genes [8, 9] as well as 59 novel families of the subclass B1 metallo-beta-lactamases (MBLs) capable of degrading carbapenems from a variety of environments [10]. HMM accurately predicts the gene fragments belonging to specific gene classes, based on evolutionarily conserved domains [11]. This leads to accurate detection of both known and previously undescribed resistance genes in genomic and metagenomic sequence data. Functional metagenomics is another strategy that has the ability to identify novel ARGs without apparent similarities to known ARGs as it is based on a functional selection of DNA fragments expressed in a surrogate host like *Escherichia coli* [12]. With such an approach, we have identified novel ARGs, including one encoding a carbapenem hydrolyzing beta-lactamase, from river sediments contaminated with drug production waste [13]. Several studies, using functional metagenomics, have reported novel resistance genes from a variety of environments like human gut, soil, and seawater [14–18].

Neither approach relies on the host-bacteria being cultivable, but both largely lack the ability to differentiate between mobilized and non-mobilized genes. The latter is a critical aspect for assessing risk associated with ARGs [19, 20]. To overcome this, we have recently used a high-throughput sequencing method for studying genes associated with integrons [21]. Class 1 integrons are frequently carried by human pathogens and very often harbor ARGs. The integrons are usually located on mobile genetic elements like plasmids and transposons, providing the ability to move across cells, strains, and species [22–24]. Using an approach of amplifying partial class 1 integrons, the fourth mobile sulfonamide resistance gene (*sul4*) was discovered together with several other novel resistance genes, including those encoding class D beta-lactamases conferring reduced susceptibility to carbapenems in *E. coli* [21].

Hospital sewage represents a collection of fecal matter of a large number of individuals including patients undergoing antibiotic treatment, together with other bacteria of environmental origin. Antibiotic residues in hospital wastewaters may reach levels that potentially could be selective for resistant strains [25–27]. Several studies have accordingly shown that hospital effluents can provide a rich variety of known ARGs and resistant enteric pathogens [28–32]. It is therefore plausible that a more explorative analysis could lead to the discovery of novel ARGs, either easily accessible to pathogens or already circulating among pathogens undetected.

Antibiotic resistance is a growing problem in India, partly due to high consumption of broad-spectrum antibiotics, including carbapenems [1]. Both clinical and environmental resistance has been on the rise in India in the last decade [33]. For example, the incidences of carbapenem resistance in *Klebsiella* isolates and the rate of methicillin-resistant *Staphylococcus aureus* (MRSA) infections have gone up in the last few years [20]. Nosocomial infections with carbapenemase-producing organisms (CPOs) are quite common in India [34], and studies have shown that hospital environments are a common reservoir of CPOs [28, 29]. A recent Indian study has shown that hospital effluent contributes to the spread of carbapenemases in the external environment [30]. For these reasons, it is possible that the Indian hospital effluent could provide a particularly rich variety of carbapenemases, including not yet characterized forms.

With this background, we aimed at identifying and characterizing novel ARGs in wastewater from an Indian hospital, particularly with regards to carbapenemases. This was achieved using two approaches—(1) investigating the antibiotic resistome and microbiota of sewage effluent collected from a large hospital in the city of Mumbai in India using Illumina HiSeq-based shotgun metagenomics and identification of novel molecular class B carbapenemases from the shotgun data using hidden Markov models, and (2) investigating novel mobile resistance genes by targeted amplicon sequencing of integron gene cassettes using a combination of short-read (Illumina MiSeq) and long-read (PacBio) sequencing technologies followed by homology searches to known ARGs. With these approaches, we detected several recently discovered resistance genes as well as genes encoding previously uncharacterized carbapenemases which we also functionally verified in *E. coli*.

## Results

Shotgun sequencing of the hospital effluent metagenome resulted in 1.72 × 10^8^ filtered reads. A total of 193,098 reads (0.11% of the total reads) matched to ARGs. Together, these accounted for 0.844 copies of ARGs per 16S rRNA gene. One hundred and twelve different mobile ARG types conferring resistance against almost all major classes of antibiotics were detected (Additional file 1: Table S1).

### Carbapenemases, class 1 integrase, and virulence genes were common in hospital effluent

**Fig. 1:**
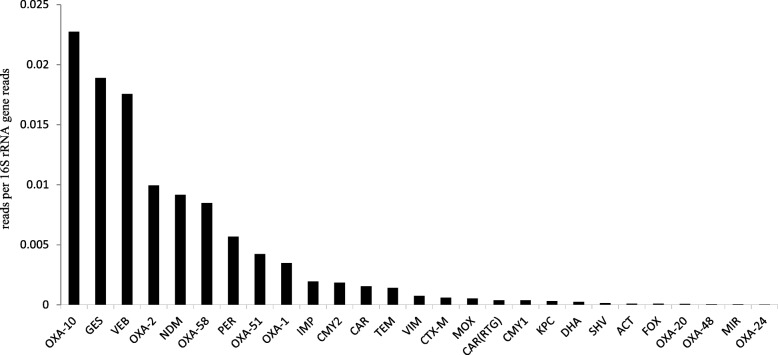
Relative abundance of different beta-lactamase genes detected in hospital effluent

The sulfonamide resistance gene *sul1* (11.4% relative abundance to 16S rRNA genes) was the most abundant resistance gene followed by the macrolide resistance gene *mphE* (11.3% relative abundance to 16S rRNA genes). Beta-lactamases were the most abundant gene class detected followed by aminoglycoside and tetracycline resistance genes (Additional file 1: Table S1). Twenty-seven different beta-lactamase genes (Fig. 1, Additional file 1: Table S1) were detected in the metagenome followed by 19 different genes conferring resistance against aminoglycosides and tetracyclines. OXA-10 and GES-type ESBLs were the dominant beta-lactamases detected in the study. The detected beta-lactamase genes also included those encoding clinically important carbapenemases, such as NDM, VIM, IMP, KPC, and OXA-48. Among the carbapenemases, NDM had the highest abundance (0.9% relative abundance to 16S rRNA genes), which corresponds to approximately 1 in 25 bacterial cells carrying NDM, if we assume on an average of 4 copies of the 16S rRNA gene per genome in the microbial community [35, 36]. OXA-58 (0.84% relative abundance to 16S rRNA genes) was the second most abundant carbapenemase, while OXA-48 and OXA-24 had the lowest abundance (0.005% and 0.0025% relative abundance to 16S rRNA genes, respectively). Among the studied mobile genetic elements, class 1 integrase (10.9% relative abundance to 16S rRNA genes) was the most common gene followed by ISCR2 and ISCR5 (Additional file 1: Table S1). Virulence genes involved in pilus formation, capsule formation, proteases, siderophore production, adhesion factors, and toxins like cytolysin and hemolysin were also detected (Additional file 2: Table S2). Some virulence genes specific to certain pathogens including enteropathogenic *E. coli* (protease *stcE*, intimin), *Salmonella typhi* (Vi antigen synthesis genes *tviBC*), *Staphylococcus aureus* (clumping factor A *clfA*), *Streptococcus pneumoniae* (fibronectin-binding protein *fbp*54), and several virulence genes for *Pseudomonas aeruginosa* were detected [37–40], indicating the presence of these pathogens in the effluent sample.

### *Acinetobacter baumannii* was abundant in hospital effluent

At the phylum level, *Proteobacteria* dominated (75% of all 16S rRNA gene reads) followed by *Bacteroidetes* (16%) and *Firmicutes* (1.75%). Within *Proteobacteria*, *Gammaproteobacteria* were the most abundant, followed by *Betaproteobacteria.* At genus level, *Acinetobacter* was found to be the most dominant genera representing 30% of the 16S rRNA reads (Additional file 3: Table S3). The abundance of the OXA-51 gene, which is a characteristic for *A. baumannii* [41], was 0.4% in relation to the total number of 16S rRNA reads. *Acinetobacter* contains 6 copies of 16S rRNA gene per genome [42]. This suggests that around 8% of total *Acinetobacter* (2.4% of the all 16S reads) detected in the samples belong to *A. baumannii*, thus, potentially representing pathogenic *Acinetobacter* strains. Further, the presence of *A. baumannii* was confirmed by alignment of the OXA-51 gene containing contigs from the assembled shotgun sequence data to *A. baumannii* genome that resulted in a perfect match. The reference-based assembly using *A. baumannii* genome sequences contained 1704 contigs (min 1 kilobases) with a total of 4.9 megabases. A single 9203 base pair long contig with 28-fold coverage matched to the original OXA-51 subsequence and was then used to identify the single closest known full genome of *A. baumannii* strain AB6200 (accession NZ_CP010397.1, 99% nucleotide identity). An alignment of the assembled contigs against this genome sequence using D-Genies (http://dgenies.toulouse.inra.fr/) [43] showed an extensive coverage of the genome by assembled contigs with high matching identity (Additional file 9: Figure S1).

### Carbapenemases and novel resistance genes, including a novel IMP variant, were detected in integron gene cassettes

**Fig. 2:**
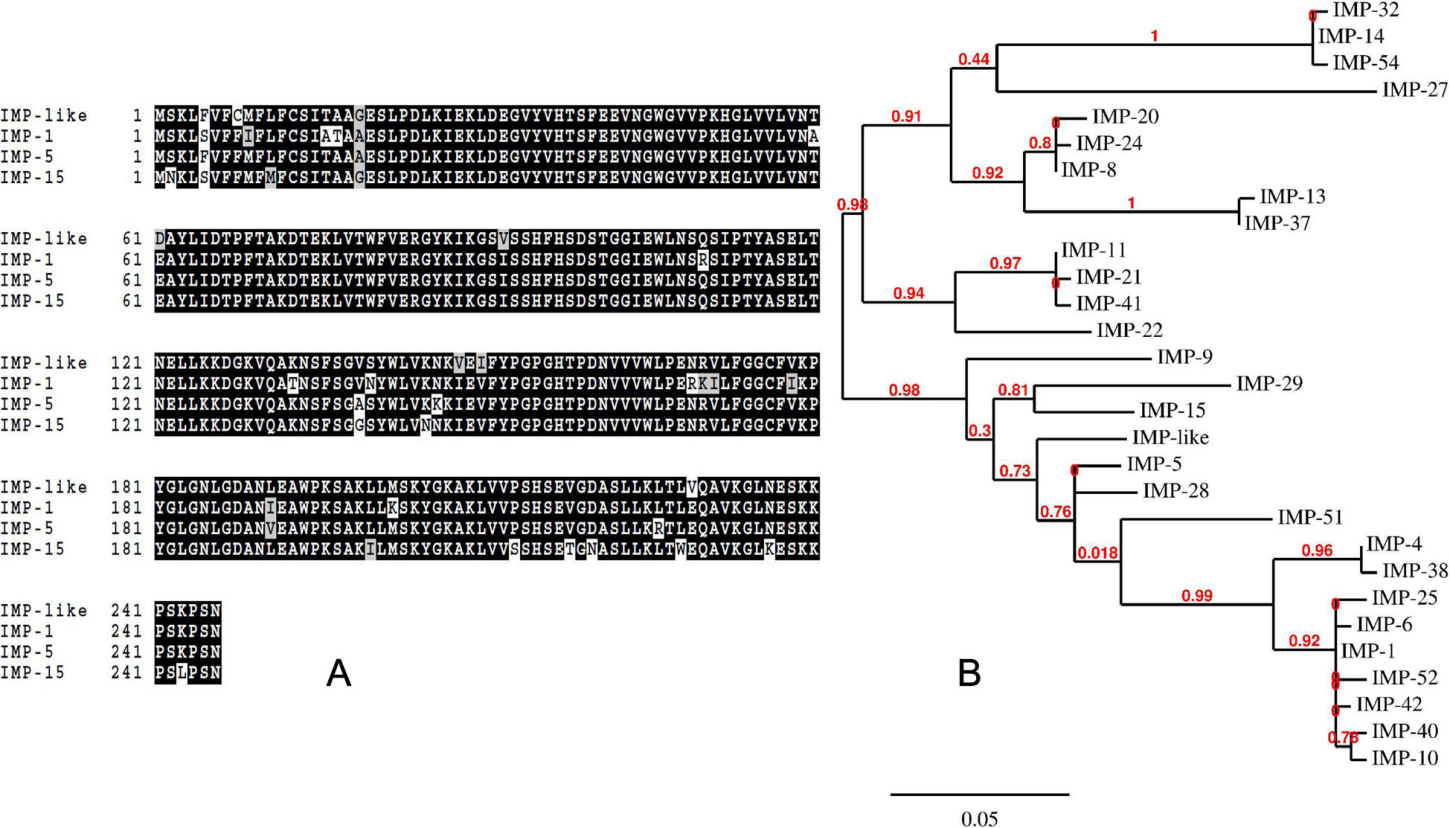
**a** Sequence alignment of novel IMP variant (proposed IMP-81) detected in our study with IMP variants 1, 5, and 15, respectively. Black color indicates consensus. Multiple sequence alignment obtained using BoxShade https://embnet.vital-it.ch/software/BOX_form.html. **b** Phylogenetic tree for IMP variants

Sequencing of gene cassette amplicons resulted in 106,851 long PacBio reads with an average length of 1.5 kilobases and 14,184,598 short Illumina reads (SRs) with a maximum length of 250 bases. A total of 250,720 open reading frames (ORFs) were identified. Clustering of all identified ORFs at 99% amino acid identity led to 6098 unique ORFs. The list of genes detected in class 1 integron gene cassettes can be found in Additional file 4: Table S4, and the list of known and putative novel ARGs detected in class 1 integron gene cassettes can be found in Additional file 5: Table S5. Several carbapenemases including DIM-1, IMP-1, IMP-15, IMP-6, VIM-2, and GES-type beta-lactamases were detected, along with several putative novel resistance genes/gene variants (Table 1). We also detected many variants of GES (GES-2, GES-4, GES-5, GES-6, GES-14) capable of carbapenem hydrolysis in gene cassettes from hospital effluent. Recently reported ARGs like *bla*_RSA1_ and *sul4* were also found as part of the gene cassettes. Interestingly, we detected a novel variant of an IMP-type carbapenemase. This novel variant (proposed-IMP-81) has 8 aa substitutions compared to the closest known variant of IMP-15 (Fig. 2). This gene provided reduced susceptibility against carbapenems when expressed in *E. coli* (Additional file 6: Table S6) and was positive for imipenem degradation in the CarbaNP test.

**Table 1:** Putative novel resistance genes and gene variants detected in integron gene cassettes

Gene name	Length (amino acid)	Closest homologue in NCBI protein database	Identity (%)	Accession number	Resistance against
aadA- like-1	288	Aminoglycoside adenyltransferase, partial [*Escherichia coli*]	89	AEG64741.1	Aminoglycosides
sat-like-1	301	Streptomycin 3-adenylyltransferase [Yersinia pestis biovar Orientalis str. IP275]	94	EDR32758.1	Aminoglycosides
aadA- like-2	287	Aminoglycoside adenyltransferase, partial (plasmid) [*Escherichia coli*]	94	AIL81899.1	Aminoglycosides
CAT-like	170	Chlor_Acetyltrans_CAT, partial [uncultured bacterium]	92	AMP56890.1	Amphenicols
*bla*_RSA1_*	270	Class A extended-spectrum beta-lactamase BEL-1 [*Pseudomonas aeruginosa*]	53	WP_063857830.1	Beta-lactams
OXA-like-1	300	Class D [uncultured bacterium]	87	AMP47162.1	Beta-lactams
OXA-like-2	266	Class D [uncultured bacterium]	91	AMP48561.1	Beta-lactams
OXA-like-3	266	Oxacillin-hydrolyzing class D beta-lactamase OXA-10 [*Gammaproteobacteria*]	92	WP_000846390.1	Beta-lactams
OXA-like-4	266	Oxacillin-hydrolyzing class D beta-lactamase OXA-119 [Pseudomonas aeruginosa]	93	WP_032490445.1	Beta-lactams
AmpC-like-1	329	Beta-lactamase [*Vibrio cholerae* non-O1/non-O139]	94	BAE71359.1	Beta-lactams
OXA-like-5	275	Oxacillin-hydrolyzing class D beta-lactamase OXA-2 [*Proteobacteria*]	95	WP_001007673.1	Beta-lactams
IMP-like-1 (IMP-81)	246	Subclass B1 metallo-beta-lactamase IMP-15 [Pseudomonas aeruginosa]	96	WP_063860575.1	Beta-lactams
qacE-like-1	110	QacE [uncultured bacterium]	81	ACN22612.1	Disinfectants
qacG-like-1	96	QacG, partial [uncultured bacterium]	90	ACS73614.1	Disinfectants
arr-like-1	150	NAD(+)--rifampin ADP-ribosyltransferase Arr-6 [*Pseudomonas aeruginosa*]	86	WP_063842214.1	Rifampicin
*sul4* ^#^	280	Dihydropteroate synthase [*Ardenticatena*]	68	CUS02277.2	Sulfonamides
Dfr-like 1	107	Dihydrofolate reductase [*Pseudomonas aeruginosa*]	88	BAT62904.1	Trimethoprim
Dfr-like 2	157	Dihydrofolate reductase, partial [*Vibrio cholerae*]	89	AAT37842.1	Trimethoprim

*The sequence is 100% identical to *bla*_RSA1_

^#^The sequence is 100% identical to *sul4*

### Novel class B carbapenemase genes outnumbered previously characterized carbapenemases in the hospital effluent microbiota

We applied a recently developed computational method based on hidden Markov models to predict novel subclass B1, B2, and B3 carbapenemases directly from the shotgun data. In total, we detected 14 unique ORFs representing subclass B1, one unique ORFs representing B2, and 12 unique ORFs representing subclass B3, respectively (Table 2). The phylogenetic trees for the subclass B1/B2 and subclass B3 genes detected are presented as Additional file 10: Figure S2 and Additional file 11: Figure S3. Out of the 27 unique ORFs, seven represented previously characterized genes, viz NDM-1, IMP-1, IMP-15, VIM-2, DIM-1, POM-1, and L1, respectively. We detected nine putative novel subclass B1, one novel subclass B2, and 10 novel subclass B3 carbapenemases, respectively. The list of these genes and the closest blast hits are presented in Table 2. The putative novel subclass B2 gene (2N30) was expressed in *E. coli*, and the CarbaNP test confirmed its ability to hydrolyze imipenem. Seven of the novel predicted B3 carbapenemases were synthesized and tested for imipenem degradation using CarbaNP test. Six out of the seven genes were positive for the test (Table 2). One of the subclass B1(1N7) protein falls in the same phylogenetic cluster as SPS-1 (Additional file 10: Figure S2). This enzyme has a substitution at position 116 (histidine at position 116 is replaced by a glycine), consistent with other enzymes belonging to this phylogenetic cluster [10].

**Table 2:** The list of MBLs detected using computational model and shotgun sequence data

Gene name	MBL subclass	Length aa	Closest homolog in NCBI protein database	% identity aa	Accession number
DIM	B1	202	Subclass B1 metallo-beta-lactamase DIM-1 [*Pseudomonas stutzeri*]	100.0	WP_063860203.1
NDM	B1	248	Metallobetalactamase NDM-1 [*Klebsiella pneumoniae*]	100.0	AGC54622.1
IMP	B1	240	Beta-lactamase IMP-1 precursor [Pseudomonas aeruginosa]	100.0	CRX26419.1
VIM	B1	248	Subclass B1 metallo-beta-lactamase VIM-2 [*Pseudomonadales*]	100.0	WP_003108247.1
IMP	B1	240	Subclass B1 metallo-beta-lactamase IMP-15 [*Pseudomonas aeruginosa*]	100.0	WP_063860575.1
1N26	B1	241	Beta-lactamase [uncultured bacterium]	74.79	ALG03680.1
1N27	B1	242	Subclass B1 metallo-beta-lactamase [bacterium 336/3]	71.97	WP_054042800.1
2N30*	B2	252	ChpA family subclass B2 metallo-beta-lactamase [*Aeromonas lacus*]	51.48	WP_033113784.1
1N32	B1	241	Hypothetical protein A3D31_07435 [*Fluviicola* sp. RIFCSPHIGHO2_02_FULL_43_260]	55.04	OGS79779.1
1N4	B1	247	Subclass B1 metallo-beta-lactamase [bacterium 336/3]	73.55	WP_054042800.1
1N59	B1	244	Hypothetical protein A2041_05420 [*Bacteroidetes bacterium* GWA2_31_9b]	52.7	OFX20903.1
1N6	B1	246	Subclass B1 metallo-beta-lactamase [bacterium 336/3]	59.5	WP_054042800.1
1N8	B1	234	Subclass B1 metallo-beta-lactamase [*Flectobacillus major*]	70.67	WP_044171073.1
1N9	B1	240	Beta-lactamase [uncultured bacterium]	66.53	ALG03680.1
1N7	B1	273	Hypothetical protein Gferi_08260 [*Geosporobacter ferrireducens*]	48.0	AOT69571.1
POM-1	B3	223	B3 beta-lactamase [*Pseudomonas otitidis*]	100.0	ADC79563.1
L-1	B3	210	LW82289.1 metallo-beta-lactamase L1 family protein [*Acinetobacter* sp. WC-743]	99.52	WP_009585815.1
3N14	B3	283	Subclass B3 metallo-beta-lactamase [*Phenylobacterium* sp. Root700]	69.58	WP_056733210.1
3N32*	B3	281	BJP_beta_lactamase [uncultured bacterium]	61.59	AIA10847.1
3N33	B3	289	BJP_beta_lactamase [uncultured bacterium]	61.4	AIA10847.1
3N40*	B3	290	Subclass B3 metallo-beta-lactamase [*Phenylobacterium* sp. Root700]	69.96	WP_056733210.1
3N51*	B3	299	Subclass B3 metallo-beta-lactamase [*Novosphingobium* sp. PP1Y]C	70.57	WP_013834039.1
3N55*	B3	284	Subclass B3 metallo-beta-lactamase [*Phenylobacterium* sp. Root700]K	70.82	WP_056733210.1
3N61*	B3	297	Subclass B3 metallo-beta-lactamase [*Croceicoccus marinus*]	57.65	WP_066847047.1
3N73^#^	B3	295	BJP_beta_lactamase [uncultured bacterium]	62.98	AIA10847.1
3N8	B3	297	Subclass B3 metallo-beta-lactamase [*Sphingomonadaceae*]E	55.85	WP_008831296.1
3N1*	B3	300	Subclass B3 metallo-beta-lactamase [Novosphingobium sp. Leaf2]	53.0	WP_056771586.1

*These genes were positive in the CarbaNP test

^#^This gene was negative in the CarbaNP test

## Discussion

In order to identify novel resistant determinants, particularly carbapenemases, we explored sewage effluent collected from a hospital in India, a country with high use of carbapenems and widespread problems with carbapenemase resistance. Using both hidden Markov models on shotgun data, as well as amplicon sequencing of integron gene cassettes, we found several novel ARGs. These include several different carbapenemases that also turned out to be functional when expressed in *E. coli*. The present study therefore expands our knowledge of novel resistance genes. The co-occurrence of pathogens and novel resistance genes to critically important antibiotics offers increased opportunities for unwanted horizontal gene transfer events. Hence, the studied hospital sewage environment appears to provide an example of the “ecological connectivity” [20] needed for genes to move across niches and environments to eventually become clinical problems.

We demonstrate the presence of a functional novel variant of a mobile IMP-type (proposed IMP-81) carbapenemase as a gene cassette within a class 1 integron. IMP-1 was the first mobile MBL detected and is one of the five major clinical carbapenemases found globally [44, 45]. Identification of this novel variant could reflect that MBLs are constantly evolving. Indeed, its presence in sewage from an Indian hospital could potentially be linked to its evolution as a result of high consumption of broad-spectrum antibiotics, including carbapenems, in India [1, 46]. Using shotgun metagenomics and computational models, we have substantially increased our understanding about the diversity of MBLs. Although several studies have detected known MBLs in hospital effluent, to the best of our knowledge, none of the studies have detected as many novel MBLs in hospital effluent to date [28, 30, 47]. We extended our previous subclass B1 model for the detection of subclass B3 MBLs and found 2 known and 10 putative novel B3 carbapenemases. Six out of seven predicted and synthesized B3 genes were functional in *E. coli*, thus demonstrating the high accuracy of our approach. Four of the predicted B3 genes belong to the same part of the phylogenetic tree as the clinically important L1 beta-lactamase and the only known version of B3 carbapenemases located on a plasmid, AIM-1 [48, 49]. Although these genes are functional in *E. coli*, the computational prediction does not provide information about their genetic context. Hence, we do not know their potential for mobility nor the host species for these genes. Further investigation of genetic context and host species identification is thus warranted.

NDM was the most abundant of the clinical carbapenemase detected in the effluent. This finding is in accordance with the clinical data on carbapenemase producing pathogens from Mumbai [50]. A recent study showed that NDM-1 is also common in hospital effluents from Delhi, India [30]. Interestingly, we detected OXA-58 as the second most abundant carbapenemase gene. OXA-58 has been found on a plasmid in *Enterobacteriaceae*, and it is a mobile carbapenemase regularly encountered in *Acinetobacter* [51, 52]. A previous study of ours showed that the abundance of OXA-58 in Indian river sediments contaminated with untreated urban waste strongly correlates with the abundance of *Acinetobacter* [53]. Similarly, the high abundance of OXA-58 in the hospital effluent detected here can be explained by a high abundance of *Acinetobacter*. The rates of *A. baumannii* nosocomial infections have risen globally in past decades [54]. *Acinetobacter* is invariably resistant to multiple antibiotics, including last resort drugs like carbapenems and colistin, making treatment difficult. *Acinetobacter* often carries conjugative plasmids bearing multidrug resistance markers and carbapenemases belonging to the OXA-type as well as MBLs. *Acinetobacter* can readily exchange these plasmids carrying drug resistance markers with members of family *Enterobacteriaceae* [51, 55].

GES-type beta-lactamases were the second most abundant ESBLs in our study, with several variants capable of low-level hydrolysis of carbapenems [56]. GES-carrying carbapenem-resistant strains have been previously isolated from hospital effluent [57]. GES-type ESBL genes are found globally and exclusively as integron gene cassettes in Gram-negative pathogens, including *P. aeruginosa*, *E. coli*, *K. pneumoniae*, and *A. baumannii* [58]. The high abundance of GES-type beta-lactamases in the shotgun dataset can at least partly be explained by high abundance of class 1 integrons in our samples. The recently discovered ESBL gene *bla*_RSA1_ was also detected in integron gene cassettes here. The *bla*_RSA1_ gene is phylogenetically close to GES-type ESBLs. The beta-lactam hydrolytic profile of the *bla*_RSA1_ protein resembled that of GES-2. Although *bla*_RSA1_ does not hydrolyze carbapenems, there is a possibility that natural mutants might occur which may possess this activity, as is the case for other GES variants [13]. The same may apply for other ESBLs, as was recently demonstrated by the discovery of a natural mutant of OXA-10 with increased carbapenemase activity in Swedish hospital effluent [59].

Sulfonamide resistance genes are common in domestic and hospital effluents [60]. Our results showed that *sul1* was the most abundant mobile resistance gene, which is in accordance with a previous study of hospital wastewater [60]. The recently described novel sulfonamide resistance gene *sul4* was detected along with the *ISCR*20 transposes as described earlier [21]. Both *sul4* and *bla*_RSA1_ were first described from river sediments contaminated with waste from drug manufacturing plants near Hyderabad, India, and concerns were raised about finding these genes in clinical isolates [13, 21]. The presence of these genes in integron gene cassettes from hospital effluent suggests that these genes are accessible to pathogens and might already have made their way into human pathogens. This finding also emphasizes the need to explore and characterize environmental ARGs, which may end up in clinics in the future.

## Conclusions

We show that explorative studies for novel antibiotic resistance determinant in hospital effluent can contribute to early identification of what may become future clinical problems. An expanded knowledge of these novel genes can facilitate actions to mitigate their potential spread in the clinic. Bacteria carrying novel ARGs, including novel carbapenemases, co-exist with pathogens, thus creating a niche where the acquisition of novel ARGs by pathogens may take place. It is also possible that such gene exchange is further boosted by the expected presence of antibiotic residues [61, 62]. If released untreated or inadequately treated, as in many low- and middle-income countries, there is increased opportunities for transmission of enteric pathogens, including resistant ones [30]. Hence, hospital effluent discharges also deserve attention from antimicrobial resistance risk management point of view.

## Methods

### Sampling, DNA extraction, and shotgun sequencing

Effluent samples were collected directly from the sewer line from a hospital in Mumbai, India, on 2 different days (30 October 2014 and 1 November 2014). Each sample comprised composite sample collected every hour (100 ml each time) during the day from 8 a.m. to 6 p.m. in a sterile plastic bottle. The sub-samples were stored at a temperature of 4 °C, mixed together and filtered on the same day using a 0.2-μM filter. DNA was extracted from the filters using QIAamp DNA Stool Mini Kit (Qiagen, Germany). The DNA was quantified using dsDNA High Sensitivity (HS) Assay kit on the Qubit® Fluorometer (Invitrogen, USA) and stored at − 20 °C. The DNA was sent for shotgun metagenomic sequencing (paired-end, 125 base pair reads) on Illumina HiSeq2500 platform at Science for Life Laboratories (Stockholm, Sweden).

### Sequence analysis of shotgun metagenomic data

The sequence analysis was performed according to the protocol described by Marathe et al. [53]. In brief, the sequences were trimmed for adaptors and quality-filtered using Trim Galore (http://www.bioinformatics.babraham.ac.uk/projects/trim_galore/) with a phred quality score of 28 and a maximum error rate (the number of errors divided by the length of the matching region) of 0.1. The quality-processed reads from the metagenomes were mapped against protein sequences from a high-quality and manually curated database of mobile ARGs and mobile genetic elements, Resqu database (Resqu database; version 1.1; 1928 Diagnostics, Gothenburg; http://www.1928diagnostics.com/resdb), which contains ARGs that have been previously reported to be horizontally transferred or carried on a mobile genetic element. Full-length coverage of the query reads was set against target resistance genes with a sequence identity threshold of 90%, and only the best hits were retrieved (options “-usearch_global -id 0.9 maxaccepts 1 -threads 16”). The list of resistance genes in the Resqu database is given in Additional file 7: Table S7. Analysis of bacterial virulence-associated genes (virulence factors) in the metagenomes was performed using a set of experimentally verified virulence factors collected from the Virulence Factor Database (http://www.mgc.ac.cn/VFs/) [63]. To characterize the overall taxonomic distribution, quality-filtered shotgun reads were used as input to extract the reads corresponding to small subunit (SSU) 16S bacterial ribosomal RNA genes from the metagenomes and assigned them to different taxonomic groups using Metaxa2 (version 2.1) with default options [64].

### PCR amplification and sequencing of integron gene cassettes

Amplification of integron gene cassettes was performed according to the protocol described by Razavi et al. [21]. In brief, integron gene cassettes were amplified from the hospital effluent DNA using three sets of primers previously described using phusion taq polymerase (thermoscientific, USA). PCR products were purified using QIAquick PCR Purification Kit (Qiagen, Germany) and quantified using the Qubit® Fluorometer (Invitrogen, USA). The purified PCR products were sent for single-molecule real-time (SMRT) sequencing technology (Pacific Biosciences) and shotgun metagenomic sequencing to produce (paired-end 250 base pair reads on the Illumina MiSeq platform) at Science for Life Laboratories in Uppsala and Stockholm, respectively.

### Sequence analysis of integron gene cassettes

Sequence analysis was performed using the method described by Razavi et al., 2017. In brief, quality-filtered PacBio reads were corrected using Illumina reads with a hybrid correction pipeline for SMRT sequencing, i.e., Proovread [65]. Reads were clustered at 100% identity using CD-HIT to remove redundancy. The open reading frames (ORFs) were predicted using Prodigal [66] and annotated through similarity searches against the NCBI non-redundant protein (nr) databases (13 April 2017). Putative novel resistance genes were identified based on their sequence identity and the length of the alignment (coverage) to known homologs genes both in the CARD (version 1.1.0, REF) and the NCBI nr protein databases. Integron gene cassettes are expected to carry a wide array of genes, including ARGs. Hence CARD and NCBI (nr), which are broader databases compared to Resqu, were used for characterizing integron gene cassettes. We classified ORFs with at least 95% identity to closest homologs in the CARD database as “known resistance genes.” Although the exact cutoff is subjective, this has been used by others in the past [67]. We classified ORFs with a best match to a resistance gene in the CARD database as “putative novel resistance genes” if they had an identity below 95% and a coverage greater than 65% [21]. The gene cassettes with known functions were clustered at 99% identity cutoff to remove redundancy using CD-HIT. HattCI was used to identify the attachment site *attC* in the reads [68].

### Prediction of class B beta-lactamases

The novel MBL genes were identified from shotgun sequence data using a computational method based on a hidden Markov model (HMM) reported recently [5, 10]. For identification of subclasses B1 and B2, the model was built using HMMER (version 3.1b1) and trained using 20 verified genes in the B1 MBLs subclass, while the model for subclass B3 was trained using 11 verified genes belonging to subclass B3. The list of the genes is represented in Additional file 8: Table S8. The subclasses B1 and B2 are rather similar while B3 is distinctly different, based on sequence identity. Also, phylogenetic evidence suggests that the resistance mechanisms of the subclasses B1, B2, and B3 may have developed independently [69, 70]. Hence, one model can detect both B1 and B2 genes, while a different model was created for B3 genes. The created and optimized models were applied directly to the fragmented data (short reads) and the fragments predicted to belong to either subclass B1, B2, or B3 were retrieved and assembled into full-length genes using SPAdes version 3.8.1 with parameter “—meta” [71]. The full-length genes were then once again subjected to the hidden Markov models, this time using a threshold score optimized for full-length genes. The genes that passed the final classification step were retrieved and clustered at a 70% amino acid sequence similarity together with previously characterized MBLs using USEARCH with parameters “-cluster_fast -id 0.7” [72]. The list of previously characterized MBLs used in clustering can be seen in Additional file 8: Table S8.

### Functional verification of candidate novel resistance genes

For each antibiotic class (except aminoglycosides, as the expression vector contained a kanamycin resistance gene), we selected one putative novel resistance gene/gene variant detected in integron gene cassettes for functional verification. Among the genes that had their best match to a known resistance gene, we chose the one with the lowest identity to a known ARG for each antibiotic class. Genes with > 95% identity to a resistance gene in CARD were not tested, with the exception of a putative novel IMP gene that had 96% identity to a known ARG. The reason for this is that carbapenemase genes are clinically very important and even small changes in MBL protein sequence may change their hydrolytic profile [73]. With regards to novel MBLs derived from the HMM analyses, seven novel genes from different clades of phylogenetic tree representing subclass B3 were selected for functional verification along with one gene representing subclass B2. The candidate novel genes were synthesized at ThermoFisher Scientific, Germany, using their GeneArt Gene Synthesis service and subcloned into the expression vector pZE21-MCS1 as described previously [13]. The plasmids containing novel resistance gene candidates were then transformed into *E. coli* C600Z1 (Expressys, Germany) by electroporation. The minimum inhibitory concentrations (MICs) of the respective antibiotics for the *E. coli* strains containing synthesized candidate novel resistance genes were determined using E-tests on Mueller-Hinton Agar plates (BioMérieux, France) with the addition of 100 ng/μl anhydrotetracycline as an inducer of the expression. *E. coli* strain containing an empty vector was used as a negative control. For verification of a putative novel IMP variant and MBLs, CarbaNP test was carried out as described earlier [74]. CarbaNP test is a biochemical test based on the detection of the acidification resulting from imipenem hydrolysis by carbapenemases [74].

### De novo assembly of *Acinetobacter baumannii* reads

Illumina reads that aligned with at least 90% identity to the nucleotide sequence of the type OXA-51 gene from *A. baumannii* strain AB030 (accession NZ_CP009257.1) were identified using BBMap (v38.32, “maxindel=2 minid=0.90 idfilter=0.90 strictmaxindel”) and were then assembled using MEGAHIT (v1.1.3, defaults) [75, 76]. The resulting 1341 base pair contig with a 26-fold coverage was searched against NCBI database using Entrez (18 Jan. 2019), and the genomes which contained highly similar homologs were selected (accessions NZ_KB849297.1, NZ_KB849308.1, NZ_CP033754.1, NZ_CP022283.1, NZ_CP027530.1, NZ_CP018332.1, NZ_CP020597.1, NZ_LN997846.1, NZ_LN865143.1). These were again used to recruit read pairs using BBMap (previous parameters) and then assembled using MEGAHIT (previous parameters).

## Additional files

Additional file 1: **Table S1.** Relative abundance (resistance gene copies per 16S rRNA gene) of antibiotic resistance genes and mobile genetic elements detected in our study. (XLSX 13 kb)

Additional file 2: **Table S2.** List of virulence genes detected in our study and their relative abundance to 16S rRNA genes. (XLSX 18 kb)

Additional file 3: **Table S3.** List of bacterial genera detected in our study and their relative abundance as a percentage of all 16S rRNA gene reads. (XLSX 48 kb)

Additional file 4: **Table S4.** List of genes detected in class 1 integron gene cassettes. (XLSX 2620 kb)

Additional file 5: **Table S5.** List of known and putative novel ARGs detected in class 1 integron gene cassettes. (XLSX 57 kb)

Additional file 6: **Table S6.** The MIC values in micrograms per milliliter for *E. coli* strains containing synthesized putative novel antibiotic resistance genes, against respective antibiotics. (DOCX 13 kb)

Additional file 7: **Table S7.** List of resistance genes in the Resqu database. (XLSX 24 kb)

Additional file 8: **Table S8.** List of resistance genes used for building model for prediction of class B MBLs and list of previously characterized genes used for clustering. (XLSX 13 kb)

Additional file 9: **Figure S1.** Alignment of assembled contigs with *A. baumannii* strain 2600 genome. (TIF 404 kb)

Additional file 10: **Figure S2.** A phylogenetic tree describing the evolutionary relationship between the subclass B1/B2 MBLs detected in this study. (PDF 117 kb)

Additional file 11: **Figure S3.** A phylogenetic tree describing the evolutionary relationship between the subclass B3 MBLs detected in this study. (PDF 43 kb)

## Data Availability

The raw sequencing data of hospital effluent have been deposited in the NCBI Sequence Read Archive (SRA) under the bio-project PRJNA497765. The gene sequences for novel MBLs have been submitted to GenBank under accession numbers MN017279-MN017299.
