# Supplementary material for: Sewage effluent from an Indian hospital harbors novel carbapenemases and integron-borne antibiotic resistance genes
Source: Microbiome. 2019 Jun 27;7:97. doi: 10.1186/s40168-019-0710-x (PMC6598227; doi:10.1186/s40168-019-0710-x)
Supplement: Supplementary file 6 — Table S6. The MIC values in micrograms per milliliter for E. coli strains containing synthesized putative novel antibiotic resistance genes, against respective antibiotics. (DOCX 13 kb) [file 40168_2019_710_MOESM6_ESM.docx]

**Supplementary table S6:** The MIC values in µg/ml for *E. coli* strains containing synthesized putative novel antibiotic resistance genes, against respective antibiotics.

| Gene name | antibiotic | MIC | MIC for control# |
| --- | --- | --- | --- |
| OXA-like 1 | ampicillin | >256 | 4 |
| OXA-like 1 | cefotaxime | 0.25 | 0.047 |
| OXa-like 1 | meropenem | 0.023 | 0.006 |
| IMP-like | ampicillin | >256 | 4 |
| IMP-like | meropenem | 0.75 | 0.012 |
| IMP-like | cefotaxime | >24 | 0.047 |
| IMP-like | doripenem | 0.25 | 0.023 |
| *sul4* | sulfamethaxozole | >1024 | 2 |
| dhfr-like 1 | trimethoprim | >32 | 0.125 |
| arr-like 1 | rifimpicin | >32 | 4 |
| *bla*_RSA1_ | ampicillin | >256 | 4 |
| *bla*_RSA1_ | cefotaxime | >24 | 0.047 |
| *bla*_RSA1_ | ertapenem | 0.008 | 0.006 |
| *bla*_RSA1_ | meropenem | 0.023 | 0.012 |

*#-E. coli* strains containing empty plasmid was used as a control.
