# Supplementary figures and images for: Sewage effluent from an Indian hospital harbors novel carbapenemases and integron-borne antibiotic resistance genes
Source: Microbiome. 2019 Jun 27;7:97. doi: 10.1186/s40168-019-0710-x (PMC6598227; doi:10.1186/s40168-019-0710-x)

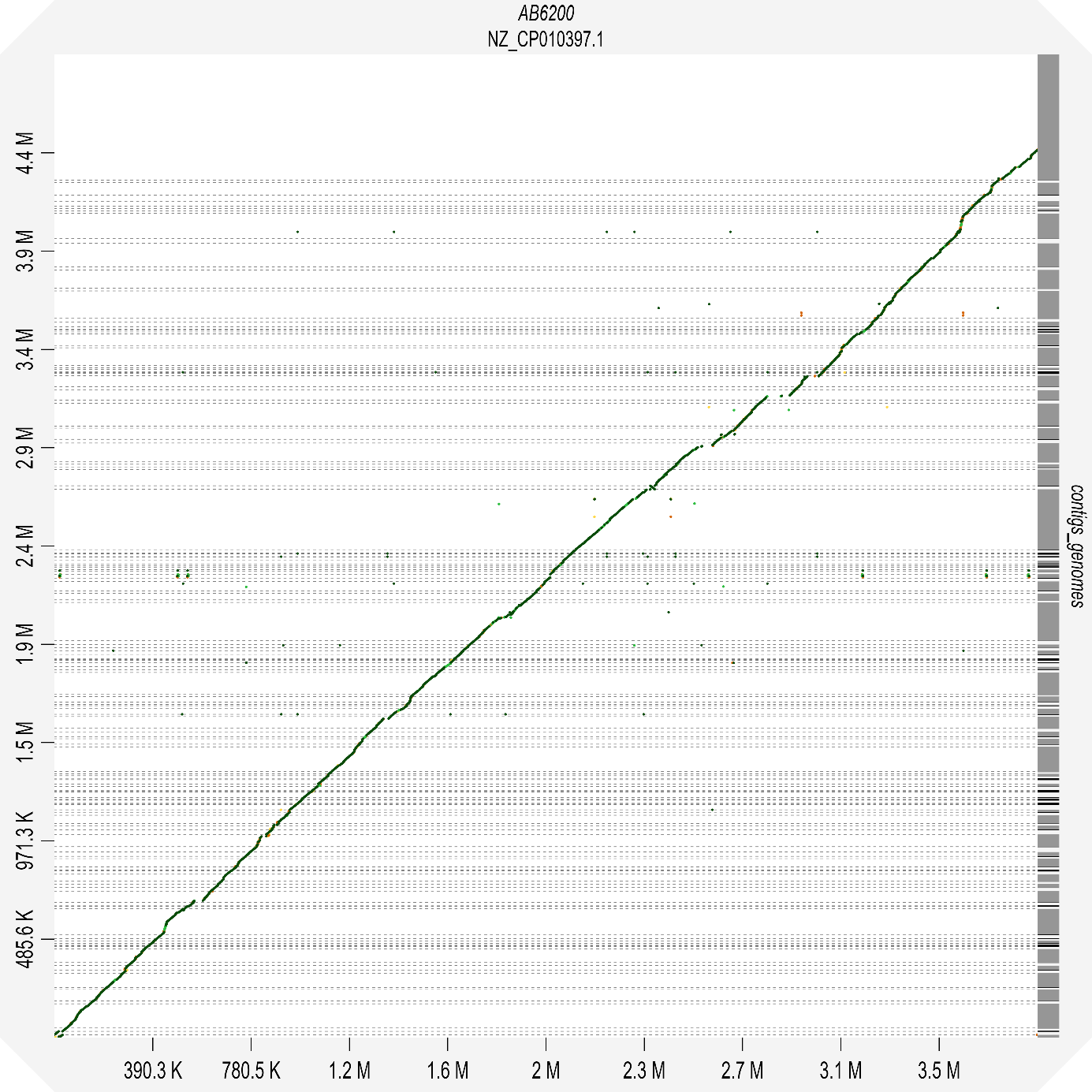

Supplement: Supplementary file 9 — Figure S1. Alignment of assembled contigs with A. baumannii strain 2600 genome. (TIF 404 kb) [file 40168_2019_710_MOESM9_ESM.tif]

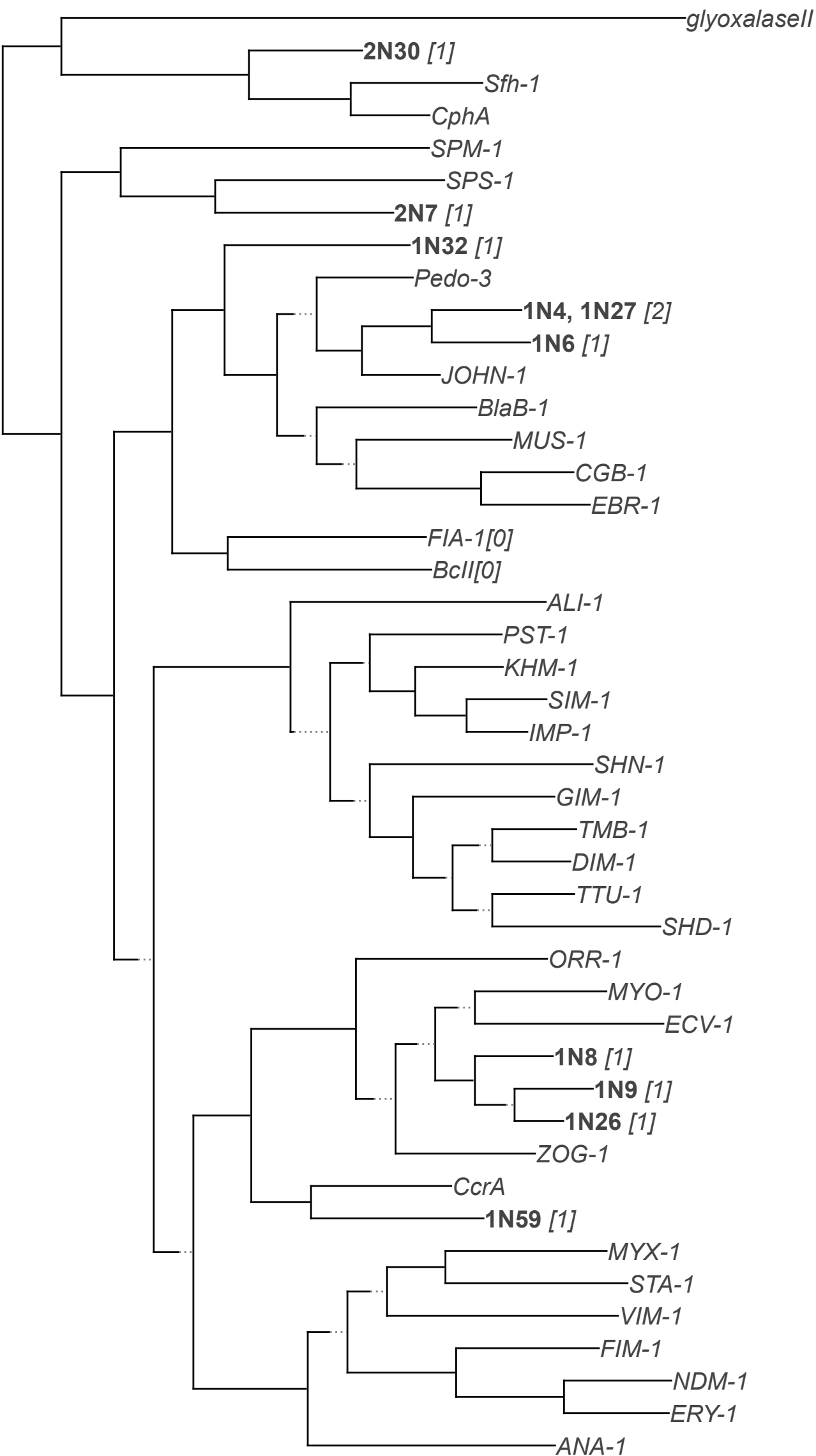

0.31

Supplement: Supplementary file 10 — Figure S2. A phylogenetic tree describing the evolutionary relationship between the subclass B1/B2 MBLs detected in this study. (PDF 117 kb) [file 40168_2019_710_MOESM10_ESM.pdf]

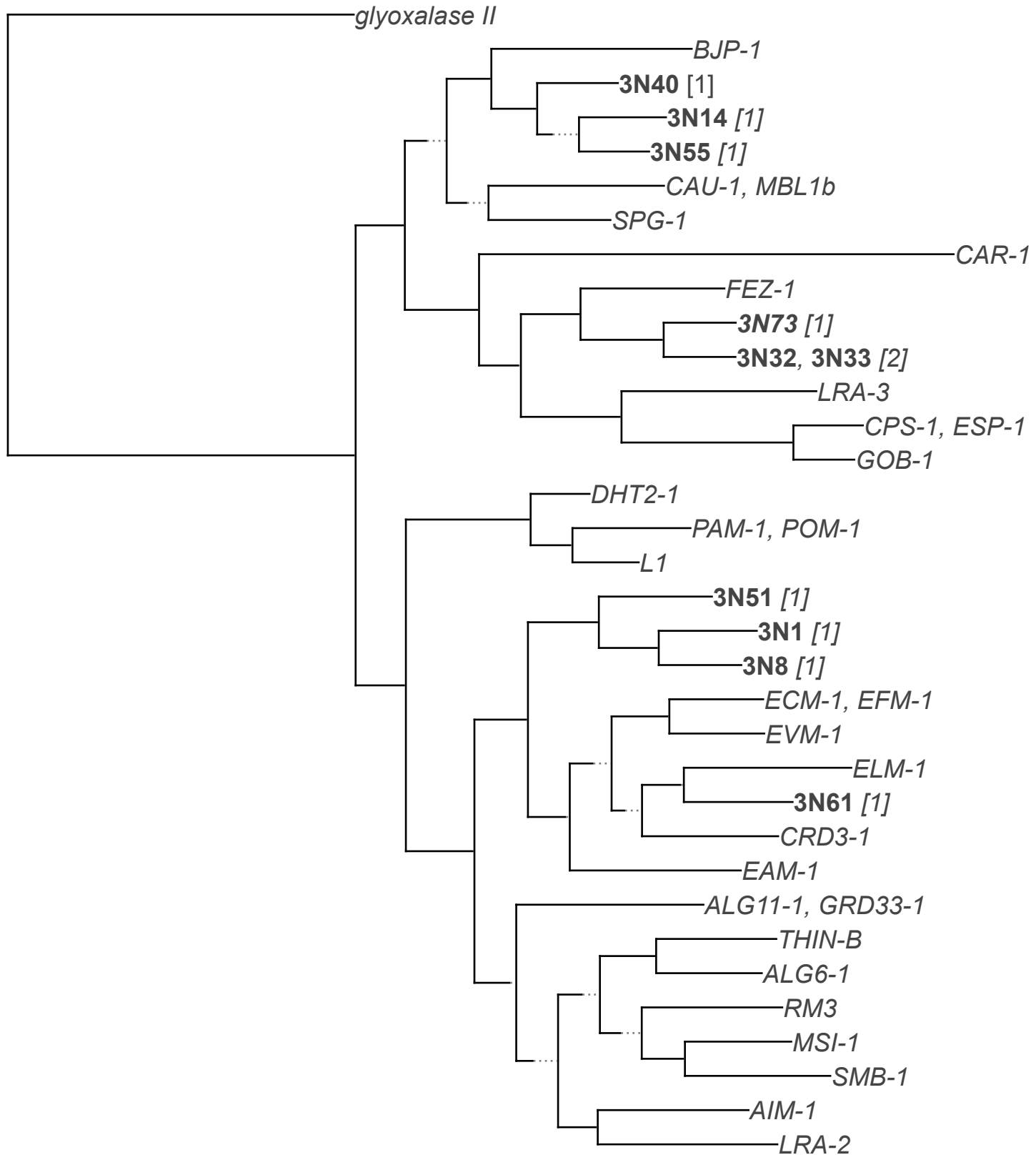

0.31

Supplement: Supplementary file 11 — Figure S3. A phylogenetic tree describing the evolutionary relationship between the subclass B3 MBLs detected in this study. (PDF 43 kb) [file 40168_2019_710_MOESM11_ESM.pdf]
